# Dichloridobis[3-methyl-4-phenyl-5-(2-pyrid­yl)-4*H*-1,2,4-triazole-κ^2^ *N* ^1^,*N* ^5^]copper(II) 3.33-hydrate

**DOI:** 10.1107/S1600536808007630

**Published:** 2008-03-29

**Authors:** Zuoxiang Wang, Yan Lan, Pingfeng Wu, Liaocheng Huang

**Affiliations:** a Ordered Matter Science Research Center, Southeast University, Nanjing 210096, People’s Republic of China

## Abstract

In the title compound, [CuCl_2_(C_14_H_12_N_4_)_2_]·3.33H_2_O, the Cu(II) atom is coordinated by two chelating 3-methyl-4-phenyl-5-(2-pyrid­yl)-1,2,4-triazole ligands and two chloride anions in a distorted octa­hedral geometry with a CuN_2_N^′^_2_Cl_2_ chromophore. The Cu atom is located on an inversion center. Two uncoordinated water mol­ecules lie on threefold rotation axes with disordered H atoms. Two hydrogen bonds are formed between the water mol­ecules, and another between water and a chlorido ligand.

## Related literature

For related literature, see: Bencini *et al.* (1987 ▶); Koningsbruggen *et al.* (1995 ▶); Moliner *et al.* (1998 ▶, 2001 ▶); Klingele & Brooker (2003 ▶); Klingele *et al.* (2005 ▶); Garcia *et al.* (1997 ▶); Lavrenova & Larionov (1998 ▶); Kahn & Martinez (1998 ▶); Koningsbruggen (2004 ▶); Matouzenko *et al.* (2004 ▶); Wang *et al.* (2005 ▶); Zhou *et al.* (2006*a* ▶,*b* ▶).
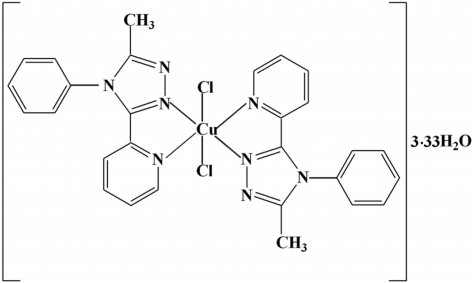


## Experimental

### 

#### Crystal data

[CuCl_2_(C_14_H_12_N_4_)_2_]·3.33H_2_O
*M* *_r_* = 667.04
Rhombohedral, 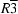

*a* = 21.5496 (13) Å
*c* = 17.619 (2) Å
*V* = 7086.0 (10) Å^3^
*Z* = 9
Mo *K*α radiation
μ = 0.91 mm^−1^
*T* = 293 (2) K
0.28 × 0.26 × 0.22 mm

#### Data collection

Bruker SMART APEX CCD diffractometer
Absorption correction: multi-scan (*SADABS*; Bruker, 2000 ▶) *T*_min_ = 0.78, *T*_max_ = 0.82
12784 measured reflections
3091 independent reflections
2155 reflections with *I* > 2σ(*I*)
*R*_int_ = 0.055

#### Refinement

*R*[*F* ^2^ > 2σ(*F* ^2^)] = 0.057
*wR*(*F* ^2^) = 0.114
*S* = 1.06
3091 reflections
194 parameters
H-atom parameters constrained
Δρ_max_ = 0.31 e Å^−3^
Δρ_min_ = −0.82 e Å^−3^

### 

Data collection: *SMART* (Bruker, 2000 ▶); cell refinement: *SAINT* (Bruker, 2000 ▶); data reduction: *SAINT*; program(s) used to solve structure: *SHELXTL* (Sheldrick, 2008 ▶); program(s) used to refine structure: *SHELXTL*; molecular graphics: *SHELXTL*; software used to prepare material for publication: *SHELXTL*.

## Supplementary Material

Crystal structure: contains datablocks I, New_Global_Publ_Block. DOI: 10.1107/S1600536808007630/cf2182sup1.cif

Structure factors: contains datablocks I. DOI: 10.1107/S1600536808007630/cf2182Isup2.hkl

Additional supplementary materials:  crystallographic information; 3D view; checkCIF report

## Figures and Tables

**Table:** 

Cu1—N4	2.006 (2)
Cu1—N7	2.023 (2)
Cu1—Cl1	2.7537 (9)

**Table:** 

N4^i^—Cu1—N7	99.70 (9)
N4—Cu1—N7	80.30 (9)
N4^i^—Cu1—Cl1	96.83 (8)
N4—Cu1—Cl1	83.17 (8)
N7—Cu1—Cl1	89.45 (7)
N7—Cu1—Cl1^i^	90.55 (7)

Symmetry code: (i) 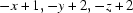.

**Table 2:** Hydrogen-bond geometry (Å, °)

*D*—H⋯*A*	*D*—H	H⋯*A*	*D*⋯*A*	*D*—H⋯*A*
O1—H1*D*⋯Cl1	0.85	2.20	3.053 (2)	179
O1—H1*A*⋯O2	0.85	2.43	2.932 (3)	119
O2—H2*B*⋯O2^ii^	0.85	1.88	2.505 (8)	130

Symmetry code: (ii) 
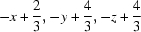
.

**Fig. 1.**
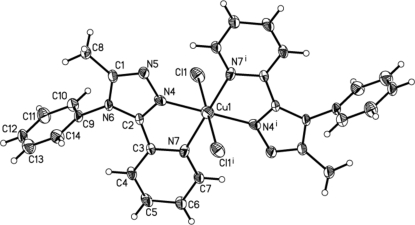
The molecular structure of the title compound with the atomic labelling. Displacement ellipsoids are shown at the 30% probability level. [Symmetry code i: -x + 1, -y + 2, -z + 2.]

## supplementary crystallographic information

### Comment

The coordination chemistry of 1,2,4-triazole derivatives has attracted great attention in recent years (Bencini *et al.*, 1987; Koningsbruggen *et al.*, 1995; Moliner *et al.*, 1998, 2001; Klingele & Brooker, 2003; Klingele *et al.*, 2005). Some spin-crossover complexes of 1,2,4-triazoles with iron(II) salts have been reported, which could be used as molecular-based memory devices, displays and optical switches (Garcia *et al.*, 1997; Lavrenova & Larionov, 1998; Kahn & Martinez, 1998; Koningsbruggen, 2004; Matouzenko *et al.*, 2004). We have synthesized some new 3,4-disubstituted-5-(2-pyridyl)-1,2,4-triazoles and their transition-metal complexes (Wang *et al.*, 2005; Zhou *et al.*, 2006*a*,b). We report here the crystal structure analysis of the title compound, (I).

The structure of (I) is shown in Fig.1. In the crystal structure, the Cu(II) atom is coordinated by two chelating 3-methyl-4-phenyl-5-(2-pyridyl)-1,2,4-triazole ligands and two chloride anions in a distorted octahedral geometry with a CuN_2_N^'^_2_Cl_2_ chromophore. Two hydrogen bonds are formed between the water molecules, and another involves the chloro ligand.

### Experimental

To a warm solution of 0.944 g 3-methyl-4-phenyl-5-(2-pyridyl)-1,2,4-triazole (4.0 mmol) in 20 ml e thanol, 0.270 g copper(II) chloride (2.0 mmol) was added. The filtrate was left to stand at room temperature for several days, and blue single crystals suitable for X-ray diffraction were collected.

### Refinement

All H atoms were located in a difference Fourier map and allowed to ride on their parent atoms at distances of 0.93 Å (aromatic), 0.96 Å (methyl) and 0.85 (water), and with *U*_iso_(H) = 1.2*U*_eq_(C) or 1.5*U*_eq_(O). O2 and O3 lie on threefold rotation axes, and accordingly, their H atoms are disordered with a partial occupancy of 1/3 and U values in the range 0.64–0.85 Å^2^.

### Crystal data

**Table:**

[CuCl_2_(C_14_H_12_N_4_)_2_]·3.33H_2_O	*Z* = 9
*M**_r_* = 667.04	*F*_000_ = 3099
Rhombohedral, *R*3	*D*_x_ = 1.407 Mg m^−^^3^
Hall symbol: -R 3	Mo *K*α radiation λ = 0.71073 Å
*a* = 21.5496 (13) Å	Cell parameters from 3501 reflections
*b* = 21.5496 (13) Å	θ = 2.5–23.9º
*c* = 17.619 (2) Å	µ = 0.91 mm^−^^1^
α = 90º	*T* = 293 (2) K
β = 90º	Polyhedron, blue
γ = 120º	0.28 × 0.26 × 0.22 mm
*V* = 7086.0 (10) Å^3^	

### Data collection

**Table:**

Bruker SMART APEX CCD diffractometer	3091 independent reflections
Radiation source: sealed tube	2155 reflections with *I* > 2σ(*I*)
Monochromator: graphite	*R*_int_ = 0.055
*T* = 293(2) K	θ_max_ = 26.0º
φ and ω scans	θ_min_ = 1.9º
Absorption correction: multi-scan(SADABS; Bruker, 2000)	*h* = −26→26
*T*_min_ = 0.78, *T*_max_ = 0.82	*k* = −21→26
12784 measured reflections	*l* = −21→19

### Refinement

**Table:**

Refinement on *F*^2^	Secondary atom site location: difference Fourier map
Least-squares matrix: full	Hydrogen site location: inferred from neighbouring sites
*R*[*F*^2^ > 2σ(*F*^2^)] = 0.057	H-atom parameters constrained
*wR*(*F*^2^) = 0.114	*w* = 1/[σ^2^(*F*_o_^2^) + (0.05*P*)^2^] where *P* = (*F*_o_^2^ + 2*F*_c_^2^)/3
*S* = 1.06	(Δ/σ)_max_ < 0.001
3091 reflections	Δρ_max_ = 0.31 e Å^−^^3^
194 parameters	Δρ_min_ = −0.82 e Å^−^^3^
Primary atom site location: structure-invariant direct methods	Extinction correction: none

### Special details

**Table:**

Geometry. All e.s.d.'s (except the e.s.d. in the dihedral angle between two l.s. planes) are estimated using the full covariance matrix. The cell e.s.d.'s are taken into account individually in the estimation of e.s.d.'s in distances, angles and torsion angles; correlations between e.s.d.'s in cell parameters are only used when they are defined by crystal symmetry. An approximate (isotropic) treatment of cell e.s.d.'s is used for estimating e.s.d.'s involving l.s. planes.
Refinement. Refinement of *F*^2^ against ALL reflections. The weighted *R*-factor *wR* and goodness of fit *S* are based on *F*^2^, conventional *R*-factors *R* are based on *F*, with *F* set to zero for negative *F*^2^. The threshold expression of *F*^2^ > σ(*F*^2^) is used only for calculating *R*-factors(gt) *etc*. and is not relevant to the choice of reflections for refinement. *R*-factors based on *F*^2^ are statistically about twice as large as those based on *F*, and *R*- factors based on ALL data will be even larger.

### Fractional atomic coordinates and isotropic or equivalent isotropic displacement parameters (Å^2^)

**Table:**

	*x*	*y*	*z*	*U*_iso_*/*U*_eq_	Occ. (<1)
Cu1	0.5000	1.0000	1.0000	0.04781 (19)	
C1	0.56211 (17)	0.87212 (17)	0.88733 (16)	0.0408 (7)	
C2	0.54547 (13)	0.96307 (14)	0.86885 (15)	0.0280 (6)	
C3	0.53495 (14)	1.02034 (15)	0.84128 (15)	0.0292 (6)	
C4	0.54676 (16)	1.04762 (16)	0.76855 (16)	0.0366 (7)	
H4	0.5626	1.0287	0.7307	0.044*	
C5	0.53496 (19)	1.10252 (19)	0.75306 (16)	0.0459 (8)	
H5	0.5416	1.1206	0.7040	0.055*	
C6	0.51311 (18)	1.13172 (19)	0.80953 (16)	0.0444 (8)	
H6	0.5053	1.1696	0.7995	0.053*	
C7	0.50315 (17)	1.10251 (16)	0.88239 (17)	0.0412 (7)	
H7	0.4894	1.1222	0.9214	0.049*	
C8	0.57362 (19)	0.81162 (17)	0.87105 (17)	0.0445 (8)	
H8A	0.6136	0.8167	0.9001	0.067*	
H8B	0.5834	0.8112	0.8179	0.067*	
H8C	0.5314	0.7675	0.8845	0.067*	
C9	0.56300 (18)	0.90787 (16)	0.74991 (16)	0.0380 (7)	
C10	0.4997 (2)	0.86341 (18)	0.71288 (19)	0.0504 (8)	
H10	0.4565	0.8395	0.7389	0.061*	
C11	0.5032 (2)	0.8556 (2)	0.6345 (2)	0.0574 (9)	
H11	0.4617	0.8262	0.6072	0.069*	
C12	0.56770 (19)	0.89132 (18)	0.59789 (17)	0.0458 (8)	
H12	0.5696	0.8859	0.5457	0.055*	
C13	0.6281 (2)	0.9340 (2)	0.6358 (2)	0.0563 (9)	
H13	0.6711	0.9579	0.6093	0.068*	
C14	0.62835 (19)	0.94346 (18)	0.71455 (19)	0.0509 (8)	
H14	0.6704	0.9722	0.7413	0.061*	
N4	0.54193 (13)	0.94977 (13)	0.94163 (13)	0.0361 (6)	
N5	0.55287 (15)	0.89213 (14)	0.95473 (14)	0.0411 (6)	
N6	0.55975 (13)	0.91589 (13)	0.83203 (13)	0.0333 (5)	
N7	0.51276 (12)	1.04744 (13)	0.89762 (13)	0.0356 (6)	
Cl1	0.37209 (5)	0.88666 (5)	0.95079 (5)	0.0581 (3)	
O1	0.38596 (12)	0.77383 (11)	0.85941 (11)	0.0424 (5)	
H1D	0.3825	0.8054	0.8850	0.051*	
H1A	0.3444	0.7409	0.8465	0.051*	
O2	0.3333	0.6667	0.7377 (2)	0.0567 (11)	
H2A	0.2895	0.6359	0.7304	0.068*	0.334
H2B	0.3504	0.6925	0.6983	0.068*	0.334
O3	0.6667	0.3333	0.70495 (19)	0.0428 (9)	
H3A	0.6650	0.3103	0.6651	0.051*	0.3333
H3C	0.6474	0.3042	0.7416	0.051*	0.3333

### Atomic displacement parameters (Å^2^)

**Table:**

	*U*^11^	*U*^22^	*U*^33^	*U*^12^	*U*^13^	*U*^23^
Cu1	0.0484 (3)	0.0501 (4)	0.0456 (4)	0.0251 (3)	0.0044 (3)	0.0055 (3)
C1	0.0531 (19)	0.0518 (18)	0.0298 (17)	0.0356 (16)	0.0098 (14)	0.0032 (14)
C2	0.0275 (14)	0.0340 (15)	0.0229 (13)	0.0158 (12)	0.0030 (11)	−0.0039 (11)
C3	0.0295 (14)	0.0369 (15)	0.0227 (13)	0.0178 (12)	0.0003 (11)	−0.0025 (11)
C4	0.0469 (17)	0.0542 (18)	0.0184 (13)	0.0326 (15)	−0.0003 (12)	−0.0004 (13)
C5	0.062 (2)	0.068 (2)	0.0205 (14)	0.0417 (19)	0.0051 (14)	0.0132 (15)
C6	0.064 (2)	0.062 (2)	0.0292 (15)	0.0477 (18)	0.0059 (15)	0.0107 (15)
C7	0.060 (2)	0.0531 (19)	0.0302 (15)	0.0429 (16)	0.0118 (14)	0.0119 (14)
C8	0.065 (2)	0.052 (2)	0.0315 (17)	0.0406 (18)	0.0071 (15)	0.0038 (14)
C9	0.064 (2)	0.0440 (17)	0.0176 (14)	0.0356 (16)	0.0047 (14)	−0.0010 (12)
C10	0.064 (2)	0.053 (2)	0.0408 (18)	0.0341 (18)	0.0118 (17)	0.0035 (16)
C11	0.062 (2)	0.070 (2)	0.0377 (19)	0.031 (2)	−0.0055 (18)	−0.0072 (18)
C12	0.070 (2)	0.057 (2)	0.0244 (16)	0.0420 (19)	0.0051 (16)	−0.0025 (14)
C13	0.063 (2)	0.068 (2)	0.042 (2)	0.036 (2)	−0.0001 (18)	0.0004 (18)
C14	0.050 (2)	0.055 (2)	0.0416 (19)	0.0214 (17)	−0.0045 (16)	−0.0022 (16)
N4	0.0456 (14)	0.0424 (14)	0.0237 (13)	0.0245 (12)	0.0007 (10)	0.0104 (10)
N5	0.0589 (17)	0.0484 (15)	0.0291 (13)	0.0367 (14)	0.0058 (12)	0.0062 (11)
N6	0.0484 (15)	0.0400 (13)	0.0197 (11)	0.0282 (12)	0.0069 (10)	−0.0004 (10)
N7	0.0401 (13)	0.0454 (14)	0.0329 (13)	0.0303 (12)	−0.0029 (11)	−0.0059 (11)
Cl1	0.0439 (5)	0.0671 (6)	0.0520 (5)	0.0191 (4)	0.0020 (4)	0.0033 (4)
O1	0.0534 (13)	0.0488 (13)	0.0327 (11)	0.0313 (11)	0.0046 (10)	0.0029 (10)
O2	0.0633 (16)	0.0633 (16)	0.043 (3)	0.0317 (8)	0.000	0.000
O3	0.0519 (14)	0.0519 (14)	0.0245 (18)	0.0259 (7)	0.000	0.000

### Geometric parameters (Å, °)

**Table:**

Cu1—N4^i^	2.006 (2)	C8—H8A	0.960
Cu1—N4	2.006 (2)	C8—H8B	0.960
Cu1—N7	2.023 (2)	C8—H8C	0.960
Cu1—N7^i^	2.023 (2)	C9—C14	1.371 (5)
Cu1—Cl1	2.7537 (9)	C9—C10	1.377 (5)
Cu1—Cl1^i^	2.7537 (9)	C9—N6	1.463 (3)
C1—N5	1.312 (4)	C10—C11	1.398 (5)
C1—N6	1.375 (4)	C10—H10	0.930
C1—C8	1.472 (4)	C11—C12	1.368 (5)
C2—N4	1.308 (3)	C11—H11	0.930
C2—N6	1.365 (3)	C12—C13	1.338 (5)
C2—C3	1.446 (4)	C12—H12	0.930
C3—N7	1.354 (3)	C13—C14	1.402 (5)
C3—C4	1.380 (4)	C13—H13	0.930
C4—C5	1.356 (4)	C14—H14	0.930
C4—H4	0.930	N4—N5	1.395 (3)
C5—C6	1.380 (4)	O1—H1D	0.850
C5—H5	0.930	O1—H1A	0.850
C6—C7	1.398 (4)	O2—H2A	0.850
C6—H6	0.930	O2—H2B	0.8499
C7—N7	1.330 (4)	O3—H3A	0.850
C7—H7	0.930	O3—H3C	0.850
			
N4^i^—Cu1—N4	180.000 (1)	C1—C8—H8B	109.5
N4^i^—Cu1—N7	99.70 (9)	H8A—C8—H8B	109.5
N4—Cu1—N7	80.30 (9)	C1—C8—H8C	109.5
N4^i^—Cu1—N7^i^	80.30 (9)	H8A—C8—H8C	109.5
N4—Cu1—N7^i^	99.70 (9)	H8B—C8—H8C	109.5
N7—Cu1—N7^i^	180.000 (1)	C14—C9—C10	123.8 (3)
N4^i^—Cu1—Cl1	96.83 (8)	C14—C9—N6	118.7 (3)
N4—Cu1—Cl1	83.17 (8)	C10—C9—N6	117.5 (3)
N7—Cu1—Cl1	89.45 (7)	C9—C10—C11	117.3 (3)
N4^i^—Cu1—Cl1^i^	83.17 (8)	C9—C10—H10	121.4
N4—Cu1—Cl1^i^	96.83 (8)	C11—C10—H10	121.4
N7—Cu1—Cl1^i^	90.55 (7)	C12—C11—C10	119.9 (3)
N7^i^—Cu1—Cl1^i^	89.45 (7)	C12—C11—H11	120.1
Cl1—Cu1—Cl1^i^	180.00 (4)	C10—C11—H11	120.1
N5—C1—N6	110.6 (3)	C13—C12—C11	121.2 (3)
N5—C1—C8	126.0 (3)	C13—C12—H12	119.4
N6—C1—C8	123.4 (3)	C11—C12—H12	119.4
N4—C2—N6	108.2 (2)	C12—C13—C14	121.7 (3)
N4—C2—C3	120.0 (2)	C12—C13—H13	119.2
N6—C2—C3	131.8 (2)	C14—C13—H13	119.2
N7—C3—C4	121.8 (3)	C9—C14—C13	116.1 (3)
N7—C3—C2	111.2 (2)	C9—C14—H14	121.9
C4—C3—C2	127.0 (2)	C13—C14—H14	121.9
C5—C4—C3	118.9 (3)	C2—N4—N5	109.9 (2)
C5—C4—H4	120.5	C2—N4—Cu1	112.15 (18)
C3—C4—H4	120.5	N5—N4—Cu1	135.90 (18)
C4—C5—C6	120.6 (3)	C1—N5—N4	105.3 (2)
C4—C5—H5	119.7	C2—N6—C1	105.9 (2)
C6—C5—H5	119.7	C2—N6—C9	126.9 (2)
C5—C6—C7	117.9 (3)	C1—N6—C9	126.8 (2)
C5—C6—H6	121.1	C7—N7—C3	118.9 (3)
C7—C6—H6	121.1	C7—N7—Cu1	126.0 (2)
N7—C7—C6	122.0 (3)	C3—N7—Cu1	115.07 (19)
N7—C7—H7	119.0	H1D—O1—H1A	109.5
C6—C7—H7	119.0	H2A—O2—H2B	109.5
C1—C8—H8A	109.5	H3A—O3—H3C	109.5

Symmetry codes: (i) −*x*+1, −*y*+2, −*z*+2.

### Hydrogen-bond geometry (Å, °)

**Table:**

*D*—H···*A*	*D*—H	H···*A*	*D*···*A*	*D*—H···*A*
O1—H1D···Cl1	0.85	2.20	3.053 (2)	179
O1—H1A···O2	0.85	2.43	2.932 (3)	119
O2—H2B···O2^ii^	0.85	1.88	2.505 (8)	130

Symmetry codes: (ii) −*x*+2/3, −*y*+4/3, −*z*+4/3.
